# A new regularized least squares support vector regression for gene selection

**DOI:** 10.1186/1471-2105-10-44

**Published:** 2009-02-03

**Authors:** Pei-Chun Chen, Su-Yun Huang, Wei J Chen, Chuhsing K Hsiao

**Affiliations:** 1 Bioinformatics and Biostatistics Core Laboratory, National Taiwan University, Taipei, Taiwan, Republic of China; 2 Institute of Statistical Science, Academia Sinica, Taipei, Taiwan, Republic of China; 3 Department of Public Health, National Taiwan University, Taipei, Taiwan, Republic of China; 4 Institute of Epidemiology, National Taiwan University, Taipei, Taiwan, Republic of China

## Abstract

**Background:**

Selection of influential genes with microarray data often faces the difficulties of a large number of genes and a relatively small group of subjects. In addition to the curse of dimensionality, many gene selection methods weight the contribution from each individual subject equally. This equal-contribution assumption cannot account for the possible dependence among subjects who associate similarly to the disease, and may restrict the selection of influential genes.

**Results:**

A novel approach to gene selection is proposed based on kernel similarities and kernel weights. We do not assume uniformity for subject contribution. Weights are calculated via regularized least squares support vector regression (RLS-SVR) of class levels on kernel similarities and are used to weight subject contribution. The cumulative sum of weighted expression levels are next ranked to select responsible genes. These procedures also work for multiclass classification. We demonstrate this algorithm on acute leukemia, colon cancer, small, round blue cell tumors of childhood, breast cancer, and lung cancer studies, using kernel Fisher discriminant analysis and support vector machines as classifiers. Other procedures are compared as well.

**Conclusion:**

This approach is easy to implement and fast in computation for both binary and multiclass problems. The gene set provided by the RLS-SVR weight-based approach contains a less number of genes, and achieves a higher accuracy than other procedures.

## Background

The development of microarray technique allows us to observe simultaneously a great number of messenger RNAs (mRNA). These microarray data can be used to cluster patients, or to determine which genes are correlated with the disease. Recently, Golub et al. [1] and Brown et al. [2] considered the classification of known disease status (called class prediction or supervised learning) using microarray data. These gene expression values are recorded from a large number of genes, where only a small subset is associated with the disease class labels. In the community of machine learning, many procedures, termed as gene selection, variable selection, or feature selection, have been developed to identify or to select a subset of genes with distinctive features. However, both the proportion of "relevant" genes and the number of tissues (subjects) are usually small, as compared to the number of genes, and thus lead to difficulties in finding a stable solution. The dimension reduction for gene selection as well as for finding influential genes is essential.

Several selection procedures utilized correlations between genes and class labels, where the correlation measure can be the Pearson correlation [3], signal-to-noise ratio [1], t-statistic [4], ratio of between-group sum of squares to within-group sum of squares [5], information-based criteria [6], information of intra-class variations and inter-class variations [7], or others (see the review paper by Saeys et al. [8]). These procedures are univariate in the sense that the correlation between genes and disease is examined for each individual gene. Although they are easy to perform, these methods consider one gene at a time and ignore the gene-gene interaction. Alternative methods are multivariate approaches, such as Markov blanket filter [9-11] and a fast correlation based filter solution [12]. These multivariate correlation methods, however, can be computationally heavy, as compared with the univariate procedures.

Different from the correlation-based approaches, other researchers assess the significance of features based on the classification accuracy, a measure of performance in classifying the testing set. Most approaches adopt support vector machines (SVMs). For instance, the sparsity of 1-norm SVM is used as an exclusion index of features [13,14]. Guyon et al. [15] introduced a backward selection method that removes at each step the gene with the smallest square weight of SVM coefficient, called recursive feature elimination (RFE). In contrast, Lee et al. [16] proposed a forward selection method, called incremental forward feature selection (IFFS). It grows from a small subset and defines a positive gap parameter indicating whether to include a new feature or not. Some genetic-algorithm-based searching approaches have been proposed as well [17,18].

Other feature selection methods utilized regression technique and/or focused on the extension to multiclass problems. Lee et al. [19] selected the influential genes via a hierarchical probit regression model. They estimated, via Markov chain Monte Carlo (MCMC) method, the probability that the *j*-th gene is influential and the probability that the *i*-th sample is a cancer tissue at a fixed gene. Sha et al. [20] have extended this approach to multiclass responses. However, no empirical result was presented. Yeung et al. [21] adopted a Bayesian model average (BMA) approach for the case of binary classes. They also discussed the extension to multiclass labels using a specially designed matrix. Similar to Lee et al. [19], Zhou et al. [22] extended the probit model into a multinomial model to select the strongest genes for multiclass problems.

In this article, we also focus on cases with multiclass responses. We select genes based on their "importance" determined by a weighted average of expression levels. If tissue samples share similar expression levels, they will be weighted similarly when calculating the importance measure for each gene. If the levels vary, then the weights will not be the same. In other words, the expressions are weighted differently. These weights are kernel weights derived from the regularized least squares support vector regression (RLS-SVR, [23]). The advantages of RLS-SVR algorithm include less computational problems caused by attributes dependence, and efficient estimates of regression coefficients indicating association between similarity measure and class response. We employ these estimates to formulate subject weights, and then proceed to selection and classification. The advantages of our approach are the flexibility in including a non-uniform weighting scheme, the ability of performing multiclass classification, and the fast and easy implementation. In the following, we introduce the proposed gene selection algorithm, discuss briefly the RLS-SVR, and outline classification rules based on the selected genes. Empirical analyses of five data sets from acute leukemia, colon cancer, small round blue tumors, breast cancer, and lung cancer studies are presented. The proposed algorithm is demonstrated and its performance is compared with the analysis conducted by others [6,7,15,16,19,21,22].

## Method

Let ${\left\{(x_{i},y_{i})\right\}}_{i=1}^{n}$ denote the training data set, where *x*_*i *_∈ X ⊂ *R*^*p *^are gene expressions and *y*_*i *_∈ {1, ..., *J*} are class memberships such as the cancer types or disease states. The traditional gene selection methods assume every sample subject (or sample tissue) with equal contribution and thus weight all samples uniformly. Our proposal considers every sample differently and assigns various weights via the RLS-SVR. In the following we introduce the principle of the proposed gene selection procedures, and illustrate the RLS-SVR algorithm for assigning weights and SVM classification.

### Principle of gene selection

Before proceeding to the procedures of gene selection, it is necessary to standardize the gene expression data. Let *A *be the collection of standardized input data with subjects by row and genes by column,

$$A={\left[\begin{array}{c} x_{1}^{T}\\ \vdots \\ x_{n}^{T} \end{array}\right]}_{n\times p},$$

where the vector *x*_*i *_denotes the standardized data of the *i*th tissue, and *A *is standardized in such a way that each row has mean zero, i.e., $\sum_{j=1}^{p}x_{ij}=0$, and variance 1, i.e., $\sum_{j=1}^{p}x_{ij}^{2}/p=1$, for all *i *= 1, ..., *n*. Earlier gene selection methods, as discussed in Introduction, regarded each tissue equally important when assessing the information of gene-disease association, and therefore used the expressions directly in their selections. Tissues of similar expressions, however, often contain some information for further investigation. For example, some "clustering" pattern may imply similar contributions to disease-gene association. Therefore, these tissues should be assigned with similar weights when computing the importance measure for each gene. In addition, the similarity between expression values can arise from similar conditions in disease stages; while the difference may be due to the different degrees of cellular mutations. In other words, the weight on each tissue should depend on its "closeness" to others and its association to disease stages. In the following, we propose a weighting scheme that accounts for the difference in contribution from different subject tissues.

The first procedure is to measure the clustering pattern between tissues via a kernel function. The kernel transformation maps data into a high dimensional space, where data with similar characters locate closely. Therefore, the kernel data ${\left[\kappa (x_{i},x_{j})\right]}_{i,j=1}^{n}$, denoted by *κ*(*A*, *A*) for short, measure the between-subject similarity (here subjects are the sample tissues). For instance, the row-vector *κ*(*x*_*i*_, *A*) = (*κ*(*x*_*i*_, *x*_1_), *κ*(*x*_*i*_, *x*_2_), ..., *κ*(*x*_*i*_, *x*_*n*_)) represents the similarity measures of gene expressions between the *i*th subject and the rest. Thus, tissues sharing similar expression levels will produce a large kernel value indicating a high similarity. Next, we determine the relative contribution of individual sample tissue by the regression coefficients of class labels on tissue similarities. This regression step is performed via RLS-SVR (more discussions about RLS-SVR and derivations are in next section) to determine the weights. The resulting *n *regression coefficients $\hat{\omega }$_1_,..., and $\hat{\omega }$_*n*_, denoted as a vector $\hat{w}$, represent the correlations between *κ*(*x*_*i*_, *A*) and *y *for *i *= 1, ..., *n*, and are regarded as the contributions of individual tissues. These numbers $\hat{w}$ are called kernel weights. The use of regression approach for classification is not new [24,25]. The fitted regression coefficients convey the information of association as well as contribution of regressors to class labels such as disease status. In the kernel data setting, the *i*th regressor is *κ*(*A*, *x*_*i*_), which records the *i*th sample tissue similarity with others. As each regressor represents a tissue effect in terms of similarity, the regression coefficients can be utilized as association measure for weighting sample tissue contribution to disease status. Combining the weights and the standardized expression data matrix *A*, we obtain a *p*-dimensional vector *β *= *A*^*T*^$\hat{\omega }$ as weighted expression genes, where the *j*th component in *β *stands for a weighted summation over all *x*_*ij*_, *i *= 1, ..., *n*, for the *j*th gene,

$$\beta_{j}={\left[\begin{array}{c} x_{1j}\\ \vdots \\ x_{nj} \end{array}\right]}^{T}\hat{w}.$$

In other words, the importance of the *j*th gene, *β*_*j*_, is a weighted average of all *n *expression levels of this gene, where the weights are tissue contributions. Ranking the *p *components by their absolute values, the resulting leading genes are candidates for the next step.

Because this kernel-weighting scheme reduces the *p *genes to a smaller intermediate candidate subset in which all expressions are close to being independent, it is useful in avoiding the curse of dimensionality and filtrating the dependence among genes. For instance, if the final search subset is of size *q *genes, we can first obtain an intermediate subset of size 10*q *genes from the original set of *p *genes, and next search the *q *candidate genes within this 10*q *intermediate subset, where both *q *and 10*q *are predetermined. Within the *q*-candidate subset, we re-weight the *n *tissues and obtain the *q *absolute weighted expression sums, denoted as {|*β*_*j*_|, *j *= 1, ..., *q*}. Define the proportion of each |*β*_*j*_| by

$$\delta_{j}=\frac{\left|\beta_{j}\right|}{\sum_{j=1}^{q}\left|\beta_{j}\right|},$$

this serves as an indication of the relative importance. If the importance of these *q *genes are about the same, the proportion of each gene, *δ*_*j*_, would be roughly 1/*q*. Therefore, a strict selection criterion would be to retain all genes with *δ*_*j *_larger than 1/*q*, and remove those with smaller *δ*_*j*_. Other less stringent criteria will be discussed in the empirical data analysis.

### Regularized least squares support vector regression and classifiers

The RLS-SVR, also known as the ridge support vector regression, is a least-squares algorithm for solving support vector regression problems [23]. Here we use RLS-SVR to estimate the kernel weights in the computation of gene importance, and next we adopt two classification methods, kernel Fisher discriminant analysis (KFDA, [26]) and support vector machine (SVM), to test the discriminant ability of the final selected genes.

By learning from the given training data, the main goal of solving a linear regression problem is to find an object function *η*(*x*), *η*(*x*) = *x*^*T*^*θ *+ *b *with slope coefficients *θ *= (*θ*_1_, ..., *θ*_*p*_)^*T *^and an intercept *b*, that can correctly predict the response, *y*, based on a new input of explanatory variables, *x*. For nonlinear extensions by support vector methods, *η *is modeled as a linear function of a nonlinear feature map, i.e., *η *= *θ*^*T*^*z *+ *b*, where *z *= Φ(*x*) is the feature map for some function Φ, which can be infinite dimensional, such that Φ(*x*)^*T*^Φ(*u*) = *κ*(*x*, *u*). The LS-SVM [23] has the decision function of the form

$$\sum_{i=1}^{n}\alpha_{i}\kappa (x,x_{i})+b,$$

where *α*_*i*_'s are the Lagrange multipliers to the optimization problem: min_*θ*,*b*,*e *_$\sum_{i=1}^{n}Ce_{i}^{2}/2+{\left\|\theta \right\|}^{2}/2$ subject to the equality constraints *e*_*i *_= *y*_*i *_- *η*(*x*_*i*_). Based on the LS-SVM formulation, here we directly model the response *η *as a kernel mixture:

(1) $$\eta (x)=\sum_{i=1}^{n}w_{i}\kappa (x,x_{i})+b,$$

where *w*_1_, *w*_2_, ..., *w*_*n *_are mixing coefficients. The least-squares approach is to minimize the square errors of regression, i.e.,

(2) $$\underset{w,b}{\min}\sum_{i=1}^{n}|y_{i}-\eta (x_{i}){|}^{2}.$$

In general, the unique solution of (2) can be determined numerically. Often the kernel predictor variables, *κ*(*x*, *x*_*i*_)'s, are highly correlated, thus, the solution of regression coefficients *w *will be unstable. This problem can be solved by adding in a penalty on the norm ||*w*|| so that no single coefficient can be too large to reveal high variance. The regression coefficients are then derived from the regularized least squares (RLS):

(3) $$\underset{w,b}{\min}\left\{\sum_{i=1}^{n}\frac{C}{2}|y_{i}-\eta (x_{i}){|}^{2}+\frac{1}{2}{\left\|w\right\|}^{2}\right\},$$

where *C *controls the trade-off between data goodness of fit and degree of regularization. The SVR here is formulated and solved in the primal space. There is a strong connection between the dual optimization and primal optimization in terms of regularized least squares [27].

In this article, the Gaussian kernel $\kappa (x,x_{i})=e^{-\gamma {\left\|x-x_{i}\right\|}^{2}}$ is used throughout. Let *κ*(*x*, *A*) be the kernel functions (*κ*(*x*, *x*_1_), ..., *κ*(*x*, *x*_*n*_)), and ${\left[\kappa (x_{i},x_{j})\right]}_{i,j=1}^{n}$, denoted as *κ*(*A*, *A*), be the kernel data matrix, where *κ*(*x*_*i*_, *x*_*j*_) represents the similarity between the *i*th and *j*th subjects. Coefficients *w *and *b *are estimated by RLS (3). The estimates of *w *are the kernel weights for subject contribution.

### Procedures

The procedures of this proposed algorithm are stated as follows:

*Step 1*. Standardize row-wise the design matrix, denoted as *A*, and calculate the *n *× *n *similarity measure matrix *κ*(*A*, *A*).

*Step 2*. Find $\hat{w}$^(1)^, the estimated regression coefficients of the regression model *y *= *κ*(*A*, *A*)*w*^(1) ^+ *b*^(1) ^by RLS-SVR, where *y *= (*y*_1_, ..., *y*_*n*_)^*T *^is the *n *× 1 vector of class memberships and *κ*(*A*, *A*) is the matrix of kernel similarity. This estimate $\hat{w}$^(1) ^is used to weight subject contribution in next step.

*Step 3*. Set a small number *q*. Let *β*^(1) ^= *A*^*T*^$\hat{w}$^(1)^, *I*_1 _be the index of the 10*q *largest |$\beta_{j}^{\left(1\right)}$| and *A*^(1) ^= {*x*_*j*_, *j *∈ *I*_1_}, where *x*_*j *_is the *j*th column of *A*, and *A*^(1) ^is an *n *× 10*q *matrix.

*Step 4*. Rerun RLS-SVR for the reduced gene data: *y *= *κ*(*A*^(1)^, *A*^(1)^)*w*^(2) ^+ *b*^(2)^. Denote the solution for *w *by $\hat{w}$^(2)^.

*Step 5*. Similar to *Step 3*, let *β*^(2) ^= *A*^(1)*T*^$\hat{w}$^(2)^. Define *I*_2 _as the index of the *q *largest |$\beta_{j}^{\left(2\right)}$| and *A*^2 ^= {$A_{j}^{\left(1\right)}$, *j *∈ *I*_2_} where $A_{j}^{\left(1\right)}$ is the *j*th column of *A*^(1)^. Note that *A*^(2) ^is an *n *× *q *matrix.

*Step 6*. Solve the regression model *y *= *κ*(*A*^(2)^, *A*^(2)^)*w *+ *b *and obtain the final estimates $\hat{w}$ and $\hat{b}$. Let *β *= (*β*_1_, ..., *β*_*q*_)^*T *^= *A*^(2)*T*^$\hat{w}$.

*Step 7*. Calculate *δ*_*j*_, *j *= 1, ..., *q*. Define *I *= {*j*, *δ*_*j *_≥ 1/*q*} and $\tilde{A}=\{A_{j}^{\left(2\right)},j\in I\}$, where $A_{j}^{\left(2\right)}$ is the *j*th column of *A*^(2)^. The resulting $\tilde{A}$ is the final expression data matrix consisting of the selected genes.

There are tuning parameter *C *and kernel parameter *γ *involved in the gene-selection procedures. In the numerical study below, we use training data cross-validation (CV) for parameters selection. Often in CV parameters selection, the search is over some lattice grid points. To speed up the CV parameter selection, we suggest to use uniform design points to replace the lattice grid points [28]. Or one may start with a crude uniform design search to locate a candidate setting of parameters and next go on a fine grid search around the candidate point. All the steps above use the same pair of (*C*, *γ*) obtained at Step 2. The gene-selection procedures have been implemented in matlab and R, and codes are available at .

## Results

### Data sets

We illustrate the proposed algorithm with data from acute leukemia [1], colon cancer [29], small, round blue cell tumors (SRBCT) [30], breast cancer [31], and lung cancer [32] studies. Once genes are selected, we conduct classifications to evaluate the performance of these genes, and compare with other existing analyses [6,7,15,16,19,21,22].

#### Acute leukemia study

Samples of the acute leukemia microarray data were taken from bone marrow or peripheral blood of patients with acute lymphoblastic leukemia (ALL) or acute myeloid leukemia (AML). The ALL group can be further divided into B-cell and T-cell ALL. In other words, the acute leukemia study can be handled as a binary-class or a three-class problem. There are 38 training samples and 34 testing samples in total. Among the 38 training cases, 27 are ALL (19 B-cell ALL and 8 T-cell ALL) and 11 are AML. In the 34 testing samples, 20 are ALL (19 B-cell ALL and 1 T-cell ALL) and 14 are AML. Each sample contains 7129 gene expressions. The 38 training samples were used in the proposed algorithm to select genes. To evaluate the performance of classification with this set of selected genes, training data were used to train the model and the 34 testing tissues were next tested to compute the accuracy.

#### Colon cancer study

For the colon cancer data set, it consists of 22 normal and 40 tumor colon tissues. There were originally 6500 genes per tissue, and 2000 expressions of the highest minimal intensity across tissues were selected. Because this data set was not split into training and testing sets, we considered all samples in the procedures of gene selection. Based on the set of selected genes, we performed a 5-fold cross-validation 10 times to examine the performance in classification.

#### Small, round blue cell tumors data

This data set contains four types of small, round blue cell tumors of childhood, including neuroblastoma, rhabdomyosarcoma, non-Hodgkin lymphoma and Ewing family of tumors. There were 63 training and 25 testing samples (5 testing samples belong to other types, and hence were removed from the testing set). The original number of genes is 6567 for each sample. Genes were excluded if their intensities are too low. The final number of genes remained for analysis was 2308. Again, only training data were used to perform gene selection.

#### Breast cancer study

This study investigated 3226 gene expression profiles to identify the gene set that can discriminate three types of breast cancer: the *BRCA1*-mutation, *BRCA2*-mutation, and sporadic cases. It is a three-class problem. There were seven samples in the first class, eight in the second, and seven in the third. All 22 samples were used to perform the procedures for gene selection, and a leave-one-out approach is adopted for classification validation.

#### Lung cancer study

This study examined the ability of discrimination with microarray data in identifying five subclasses of human lung carcinomas, including adenocarcinomas, squamous cell lung carcinomas, pulmonary carcinoids, small-cell lung carcinomas cases, and normal lung specimens. A total of 203 tissues were collected and there were 139, 21, 20, 6, and 17 samples in these five classes, respectively. The 3312 most variably expressed genes among 12600 transcript sequences were included in the data. Again, all samples were used in the procedures of gene selection, and a 5-fold cross-validation is performed 10 times to evaluate classification accuracy.

### Classification methods

To evaluate the performance of the proposed gene selection algorithm, we conduct the classification using only selected genes. Here, two classifiers are utilized, the kernel Fisher discriminant analysis (KFDA, [26]) and SVM (a smoothing SVM algorithm [33] is adopted for solving SVM solutions in our data analysis). When it comes to multiclass problems, KFDA can be applied directly; while SVM adopts the winner-takes-all in one-against-one voting. The resulting accuracy is compared with others, denoted as BVS (Bayesian variable selection) [19], BMA (Bayesian model average) [21], SGS1 and SGS2 (stable gene selection) methods based on two ranking scores [7], IFFS (incremental forward feature selection) [16], SVM-RFE (SVM recursive feature elimination) [15], EB (entropy-based) [6], and MBGS (multiclass Bayesian gene selection) [22], respectively. The SVM-RFE, BVS and IFFS can only deal with binary-class problems. For IFFS, Lee et al. [16] applied the directed acyclic graph model and converted this problem into two binary classification procedures. For instance, for the three-class leukemia, IFFS solves a two-step classification problem. The first step is to split ALL and AML (there are 14 genes selected in this step), and the second step is to further classify B-cell from T-cell within the ALL class (there are 9 genes selected in this step using the remaining 7115 genes). Furthermore, if the list of selected genes was provided in the above references, we perform the KFDA and SVM classifications, respectively, to compare the performance of various selection sets.

### Selected genes and classification ability

#### Acute leukemia study

Tables 1 and 2 list the selected final *q *genes in the candidate subset of leukemia data with two classes and three classes, respectively. Based on earlier analyses of the same data (SVM-REF by [15], and BVS by [19]), we assume the number of responsible genes is no larger than 10 and set *q *= 10 here. In Tables 1 and 2, the first column represents the absolute weighted sum, denoted by |*β*_*j*_|, of gene expressions for the *q *genes, where the sum is taken over all weighted subjects in the training set. The second column lists *δ*_*j*_, the proportion of |*β*_*j *_| in all *q *|*β*|'s. The third column is the cumulative sum of proportions, i.e., $\sum_{j}^{l}\delta_{j}$, *l *= 1, ..., *q*. The indices of these *q *genes in the original data and gene descriptions are listed in the last column. When 10 is determined *a priori *for the size of influential genes, these 10 genes ought to be reported. Alternatively, if one considers this set not small enough, a threshold of 1/*q *can be adopted. For instance, the top 4 genes in Table 1 and the top 5 genes in Table 2 all correspond to *δ*_*j *_≥ 1/10. This choice, however, usually results in a small set of candidate genes. Other set of a moderate size can include j* genes, where $\sum_{j=1}^{j^{*}}\delta_{j}$ ≥ 80%, such as the first 7 genes in both Tables 1 and 2. In the following analysis, we select genes based on the strict 1/*q *criterion, the intermediate 80% threshold, and the largest set of all *q *genes, respectively; and we examine, for each selection criterion, the corresponding classification accuracy. Results from others are also listed for comparison [6,15,16,19,21].

**Table 1:** The gene weighted sums, proportions, cumulative proportions, and corresponding gene numbers of the selected genes in acute leukemia data with two classes

weighted sum |*β*_*j*_|	proportion *δ*_*j*_	Cumulative proportions	gene number	description [1]
150.6797	0.1847	0.1847	6201	interleukin-8 precursor
125.3594	0.1536	0.3383	1882	CST3 Cystatin C (amyloid angiopathy and cerebral hemorrhage)
117.9711	0.1446	0.4829	2402	Azurocidin gene
92.7434	**0.1137**	0.5966	5552	probable G protein-coupled receptor LCR1 homolog
72.3649	0.0887	0.6853	1779	MPO Myeloperoxidase
69.6762	0.0854	0.7707	6181	PTMA gene extracted from Human prothymosin alpha mRNA
64.7264	0.0793	**0.8500**	1763	Thymosin beta-4 mRNA
61.0759	0.0749	0.9249	2345	G-gamma globin gene extracted from H. sapiens G-gamma globin and A-gamma globin genes's
55.6241	0.0682	0.9931	5308	GDP-dissociation inhibitor protein (Ly-GDI) mRNA
5.6697	0.0069	1	5648	HLA-B null allele mRNA

**Table 2:** The gene weighted sums, proportions, cumulative proportions, and corresponding gene numbers of the selected genes in acute leukemia data with three classes

weighted sum |*β*_*j*_|	proportion *δ*_*j*_	cumulative proportions	gene number	description [1]
206.1576	0.1583	0.1583	6201	interleukin-8 precursor
196.3753	0.1508	0.3091	1674	FTL Ferritin, light polypeptide
155.8362	0.1196	0.4287	1882	CST3 Cystatin C (amyloid angiopathy and cerebral hemorrhage)
143.1404	0.1099	0.5386	5552	probable G protein-coupled receptor LCR1 homolog
141.0207	**0.1083**	0.6469	2402	Azurocidin gene
120.1933	0.0923	0.7392	6209	VIM Vimentin
112.4293	0.0863	**0.8255**	4017	HLA class II histocompatibility antigen, DR alpha chain precursor
96.7316	0.0743	0.8998	5716	RPS3 Ribosomal protein S3
83.2569	0.0639	0.9637	1779	MPO Myeloperoxidase
47.4576	0.0364	1	5648	HLA-B null allele mRNA

The upper half in Table 3 is binary-class and the lower half is for three-class. The table is sub-divided into three parts, A, B, and C, where part A includes results from our RLS-SVR approach and the corresponding classification accuracy, B includes other gene selection methods with the KFDA and SVM classifiers, respectively, and C simply lists the reported results in other works. For example, the lists of selected genes were provided based on BVS [19] and BMA [21], thus the same lists were used to classify the testing cases with KFDA or SVM in part B. In addition, we apply the stable gene selection methods (SGS1 and SGS2 [7]) to select 10 genes and then classify with KFDA or SVM for comparison (part B). In contrast, the set based on IFFS [16] was not provided and therefore we report only the accuracy in part C. For the binary-class in leukemia data, when the strict 1/*q *criterion is adopted, the RLS-SVR selects 4 genes and both KFDA and SVM attain an accuracy of 0.9412, same as that of BMA with 20 selected genes (there is only one gene in common). Using the 80% threshold, the proposed algorithm selects 7 genes and both KFDA and SVM attain an accuracy of 1; while IFFS takes 14 genes to reach the same accuracy. If all *q *(*q *= 10) genes are selected, both classifiers reach accuracy of 1; while SGS1 and SGS2 achieve less accurate results.

**Table 3:** Testing accuracies under different procedures for the acute leukemia data

Binary classes			
Procedures	Classifier	No. of genes	Accuracy

A: Proposed selection and criterion			
RLS-SVR			
*δ*_*j *_≥ 1/*q*	+KFDA	4	0.9412
	+SVM	4	0.9412
∑ *δ*_*j *_≥ 80%	+KFDA	7	1
	+SVM	7	1
*q *genes	+KFDA	10	1
	+SVM	10	1
			
B: Other selection procedures			
BVS	+KFDA	5	0.9706
	+SVM	5	0.9706
BMA	+KFDA	20	1
	+SVM	20	1
SGS1	+KFDA	10	0.9118
	+SVM	10	0.9118
SGS2	+KFDA	10	0.9412
	+SVM	10	0.9412
			
C: Selection and classification together			
	IFFS	14	1
	SVM-RFE	8	1
	BVS	5	0.9706
	BMA	20	0.9412

Three classes			

Procedures	Classifier	No. of genes	Accuracy

A: Proposed selection and criterion			
RLS-SVR			
*δ*_*j *_≥ 1/*q*	+KFDA	5	0.7353
	+SVM	5	0.9118
∑ *δ*_*j *_≥ 80%	+KFDA	7	1
	+SVM	7	1
*q *genes	+KFDA	10	0.9706
	+SVM	10	0.9412
			
B: Other selection procedures			
BMA	+KFDA	15	0.9706
	+SVM	15	0.9706
SGS1	+KFDA	10	0.9118
	+SVM	10	0.8529
SGS2	+KFDA	10	0.8824
	+SVM	10	0.8529
			
C: Selection and classification together			
	IFFS	23	1
	BMA	15	0.9706

The lower half of Table 3 displays the accuracy for the three-class leukemia classification. The strict 1/*q *and 80%-cutoff criteria select 5 and 7 genes, respectively. Both KFDA and SVM classification rule with 7 selected genes reach an accuracy of 1. With the same classifiers KFDA and SVM, other gene selection procedures, BMA, SGS1, and SGS2 achieve less accuracy with more genes. When considering selection and classification together, IFFS and BMA attain the same or higher accuracy, but require more genes (23 for IFFS and 15 for BMA). In the three-class case, RLS-SVR+KFDA and RLS-SVR+SVM outperform the rest, since they reach the best accuracy with a much less number of genes than others. It is noticeable that our method does not depend on the data structure, and its computation is easy and fast. In contrast, IFFS and SVM-REF require iterations, and BVS and BMA involve the simulation of posterior samples from MCMC.

#### Colon cancer study

Similar to Tables 1 and 2, Table 4 lists the information of the *q *candidate genes of the colon cancer data. Again, we let *q *= 10 based on the information from earlier analysis in [15,16]. Here, the threshold 1/10 = 0.1 leads to 4 genes in the final model; while 80% threshold selects 5 genes. The accuracies of colon cancer in Table 5 are mean accuracies of 10 replicate runs of a random 5-fold partition for cross-validation and the last column contains the standard deviations of accuracies in these 10 replicate runs. The best accuracy is 0.94 by RLS-SVR with KFDA using 10 genes. It is higher than SGS and other methods. SVM-RFE was conducted based on one particular split of the 62 samples into 31 training and 31 testing sets and the accuracy is 0.9032; and EB adopted the leave-one-out cross-validation with accuracy 0.919.

**Table 4:** The gene weighted sums, proportions, cumulative proportions, and corresponding gene numbers of the selected genes in colon cancer data

weighted sum |*β*_*j*_|	Proportion *δ*_*j*_	cumulative proportions	gene number	description [29]
33.2835	0.2522	0.2522	164	interferon-inducible protein 1-8D (human); contains MSR1 repetitive element
28.4860	0.2158	0.4860	1378	80.7 KD alpha trans-inducing protein (Bovine herpesvirus type 1)
21.0143	0.1592	0.6272	115	H. sapiens p27 mRNA
13.9334	**0.1056**	0.7328	249	human desmin gene, complete cds.
10.4369	0.0791	**0.8119**	13	H. sapiens ACTB mRNA for mutant beta-actin (beta'-actin)
8.2575	0.0626	0.8745	16	human tra1 mRNA for human homologue of murine tumor rejection antigen gp96
5.9915	0.0454	0.9199	33	40S robosomal protein S24 (human)
5.8151	0.0441	0.9640	167	IG lambda chain C regions (human)
3.7171	0.0282	0.9922	14	myosin light chain ALKALI, smooth-muscle iosform (human)
1.0403	0 0079	1	44	ubiquitin (human)

**Table 5:** Testing accuracies under different procedures for colon cancer data

Procedures	Classifier	No. of genes	Accuracy	SD
A: Proposed selection and criterion				
RLS-SVR				
*δ*_*j *_≥ 1/*q*	+KFDA	4	0.9250	0.0083
	+SVM	4	0.9067	0.0082
∑ *δ*_*j *_≥ 80%	+KFDA	5	0.9200	0.0163
	+SVM	5	0.9183	0.0157
*q *genes	+KFDA	10	0.9400	0.0186
	+SVM	10	0.9300	0.0167
				
B: Other selection procedures				
EB	+KFDA	9	0.9283	0.0076
	+SVM	9	0.9200	0.0194
SGS1	+KFDA	10	0.925	0.0083
	+SVM	10	0.9100	0.0153
SGS2	+KFDA	10	0.9283	0.0076
	+SVM	10	0.9100	0.0186
				
C: Selection and classification together				
	IFFS	5	0.8806	0.0167
	SVM-RFE	8	0.9032	n.a.
	EB	9	0.919	n.a.

#### Small, round blue cell tumors data

The information of *q*-candidate subset for the SRBCT data is in Table 6. The numbers of selected genes are 2 and 8 with the threshold levels 0.1 and 80%, respectively. The best accuracy in Table 7 is 1 with 10 genes by RLS-SVR with either KFDA or SVM. Note that it takes 14 genes for EB to reach the same accuracy.

**Table 6:** The gene weighted sums, proportions, cumulative proportions, and corresponding gene numbers of the selected genes in SRBCT data

weighted sum |*β*_*j*_|	proportion *δ*_*j*_	cumulative proportions	gene number	description [30]
366.2124	0.2283	0.2283	509	human DNA for insulin-like growth factor II (IGF-2); exon 7 and additional ORF
293.0313	**0.1727**	0.4110	187	insulin-like growth factor 2 (somatomedin A)
139.3697	0.0869	0.4979	246	caveolin 1, caveolae protein, 22 kD
130.3774	0.0813	0.5792	1955	fibroblast growth factor receptor 4
120.8319	0.0753	0.6545	1645	olfactomedinrelated ER localized protein
118.9978	0.0742	0.7287	545	antigen identified by monoclonal antibodies 12E7, F21 and O13
110.2948	0.0688	0.7975	1954	follicular lymphoma variant translocation 1
109.6586	0.0684	**0.8659**	1389	Fc fragment of IgG, receptor, transporter, alpha
108.1788	0.0674	0.9333	1372	nucleolin
107.1303	0.0667	1	430	

**Table 7:** Testing accuracies under different procedures for SRBCT data

Procedures	Classifier	No. of genes	Accuracy
A: Proposed selection and criterion			
RLS-SVR			
*δ*_*j *_≥ 1/*q*	+KFDA	2	0.6
	+SVM	2	0.55
∑ *δ*_*j *_≥ 80%	+KFDA	8	0.95
	+SVM	8	0.95
*q *genes	+KFDA	10	1
	+SVM	10	1
			
B: Other selection procedures			
EB	+KFDA	14	1
	+SVM	14	1
SGS1	+KFDA	10	0.8
	+SVM	10	0.7
SGS2	+KFDA	10	0.85
	+SVM	10	0.85
			
C: Selection and classification together			
	EB	14	1

#### Breast cancer

Table 8 states the information of the *q *candidate genes for the breast cancer study, and Table 9 contains classification accuracies with selected genes. There are 4 and 7 genes selected under the 0.1 and 80% thresholds, respectively. The best accuracy is 0.9545 (only one is misclassified) based on 10 genes. MBGS attains the highest accuracy, while results of SGS with two classifiers are similar to ours. Since BMA selects the most significant genes within each training set (BMA also adopts leave-one-out cross-validation), different genes are selected in different training validation sets (13–18 genes).

**Table 8:** The gene weighted sums, proportions, cumulative proportions, and corresponding gene numbers of the selected genes in breast cancer data

weighted sum |*β*_*j*_|	proportion *δ*_*j*_	cumulative proportions	gene number
68.8881	0.1897	0.1897	422
49.4341	0.1361	0.3258	2886
45.0788	0.1241	0.4499	1612
42.2519	0.1163	0.5662	114
39.0654	**0.1076**	0.6738	1066
29.6513	0.0816	0.7554	3023
25.4254	0.0700	**0.8254**	719
25.0111	0.0689	0.8943	1084
20.1092	0.0554	0.9496	497
18.2996	0.0504	1	1561

**Table 9:** Testing accuracies under different procedures for breast cancer data

Procedures	Classifier	No. of genes	Accuracy
A: Proposed selection and criterion			
RLS-SVR			
*δ*_*j *_≥ 1/*q*	+KFDA	5	0.9091
	+SVM	5	0.9545
∑ *δ*_*j *_≥ 80%	+KFDA	7	0.9091
	+SVM	7	0.9545
*q *genes	+KFDA	10	0.9545
	+SVM	10	0.9545
			
B: Other selection procedures			
MBGS	+KFDA	10	0.9545
	+SVM	10	1
SGS1	+KFDA	10	0.9091
	+SVM	10	0.9545
SGS2	+KFDA	10	0.9091
	+SVM	10	0.9545
			
C: Selection and classification together			
	BMA	13–18	0.7273
	MBGS	10	1

#### Lung cancer

The information of *q *candidate genes and the classification results for the lung cancer data are listed in Tables 10 and 11, respectively. Five and seven genes are selected under the 0.1 and 80% criteria, respectively. The best result is 0.9222 using 10 genes with SVM. Both SGS1 and SGS2 can attain better accuracy if more genes (98 here) are included.

**Table 10:** The gene weighted sums, proportions, cumulative proportions, and corresponding gene numbers of the selected genes in lung cancer data

weighted sum |*β*_*j*_|	proportion *δ*_*j*_	cumulative proportions	gene number	description [32]
211.3186	0.1600	0.1600	732	GRO2 oncogene
208.0781	0.1576	0.3176	2722	ligand of neuronal nitric oxide synthase with carboxyl-terminal PDZ domain
191.642	0.1451	0.46278	2194	fatty acid binding protein 7, brain
158.3931	0.1200	0.5827	3243	bridging integrator 1
142.839	**0.1082**	0.6909	2010	progesterone binding protein
121.546	0.0921	0.7830	2096	interferon regulatory factor 3
106.1448	0.0804	**0.8634**	1881	occludin
102.9994	0.0780	0.9414	2987	apoptosis-associated tyrosine kinase
46.473	0.0352	0.9766	215	ribonuclease, RNase A family, 1 (pancreatic)
30.9358	0.0234	1	270	UNC13 (C. elegans)-like

**Table 11:** Testing accuracies under different procedures for lung cancer data

Procedures	Classifier	No. of genes	Accuracy	SD
A: Proposed selection and criterion				
RLS-SVR				
*δ*_*j *_≥ 1/*q*	+KFDA	5	0.903	0.0082
	+SVM	5	0.9051	0.0111
∑ *δ*_*j *_≥ 80%	+KFDA	7	0.9179	0.0059
	+SVM	7	0.9097	0.0065
*q *genes	+KFDA	10	0.9222	0.009
	+SVM	10	0.9071	0.0104
				
B: Other selection procedures				
SGS1	+KFDA	10	0.9005	0.0062
	+SVM	10	0.9005	0.0052
SGS2	+KFDA	10	0.8077	0.0164
	+SVM	10	0.8513	0.0015
				
C: Selection and classification together				
	SGS1	98	0.938	n.a.
	SGS2	99	0.931	n.a.

## Discussions

We propose in this article a new algorithm that identifies influential genes with rich information for classification. This approach allows the collected tissues to provide different strength of association with the disease. In other words, patients sharing similar gene expressions contribute in a similar way. The similarity between tissues is quantified via kernel functions, and RLS-SVR is applied to compute the kernel weights for tissue contribution. Genes are then selected based on their weighted expression sums. The results of empirical data analysis show that the proposed selection procedure performs better in the sense that it attains a higher accuracy based on fewer genes. Furthermore, the proposed gene selection method is not restricted to binary-class problems. It handles the multiclass responses directly. Although Lee et al. [16] dealt with the 3-type leukemia case, their method assumed the knowledge of a hierarchical structure of the three types of leukemia. This hierarchy property may not be common for other multiclass problems; and if it is, the knowledge may not be known *a priori*. When the number of genes increases, the computation of BMA [21], EB [6], and MBGS [22] become heavy and some pre-selection process may be needed. Yang et al. proposed two methods to rank the genes [7]. Their algorithms are fast, but require more genes to achieve a higher classification accuracy. In contrast, the implementation of our proposed procedures is easy, fast and accurate. In our algorithm, the most intensive computation involves solving the inverse of an *n *× *n *matrix in regression. Since *n *is usually small, there is no obvious computational load. Furthermore, other approaches often rely on iterations to find the ranking orders of genes; while our SVR-weight based procedures require only one run of seven steps.

**Figure 1:**
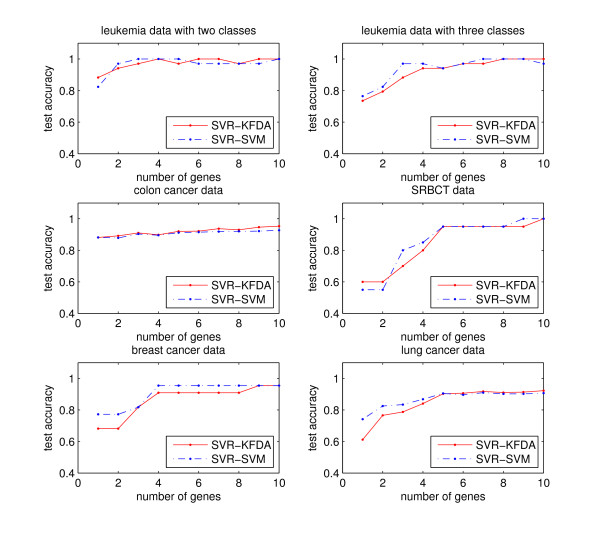
**Accuracies with respect to different numbers of genes**.

There are several issues to be discussed. First, we have set *q *= 10 in our experimental studies, and reduce from an intermediate subset of size 10*q *genes to a candidate subset of size *q*. We assign 10 for *q *under the assumption that no more than 10 genes will be included in further investigations. When other information is available, this value can be determined with ease. In our experience, the number 10*q *for the size of an intermediate set is fairly robust. Other choices do not alter the results much. Varying this number only changes slightly the order of genes in the final step. Figure 1 represents the accuracies of the five data sets with *q *genes, where *q *= 1, 2, ..., 10, respectively. The accuracy increases with the number of genes and remains stable near *q *= 10. Hence, setting 10 for *q *may be large enough to capture the influential genes. Second, as we have pointed out in previous sections, the proportion *δ*_*j *_is helpful in determining the final number of selected genes. The threshold for the number of genes to be selected can be set at different levels. If the researcher prefers a parsimonious model, he or she can set the cutting point at 1/*q *for *δ*_*j *_. If more information is desired, the value can be set at the 80% cutoff, or one can simply include all *q *genes. It can be seen from Figure 1 that the accuracy with respect to the 80% cutoff is close to that with all *q *genes. In unreported analyses, we also tried 75% and 90% as the threshold levels and have obtained similar results. Finally, we define in this article the subject weights via regressing class labels on kernel data. The class labels are denoted as 1, 2, ..., and *J*, which can be replaced by other representations. For instance, the optimal scores [25] of the first leading component would be a good choice. This may improve the performance of regularization least squares regressions. Further investigations are worth pursuing.

## Conclusion

In conclusion, with unequal kernel weights on tissues, the proposed gene selection algorithm can detect the most influential genes and obtain a higher accuracy with a less number of genes. In addition, no classifier is involved during the search of significant genes. In other words, the selected genes will not depend on or be restricted to the classifiers. For instance, the accuracies under RLS-SVR+KFDA and RLS-SVR+SVM are quite similar, which supports that the selected genes are important regardless of the classifier.

## Authors' contributions

PCC implemented the approach, coded the procedures, and prepared analysis and results. SYH was responsible for the development of the procedures, and suggested the kernel analysis and classification procedures. WJC suggested the directions for empirical analysis and for gene selections. CKH was responsible for the rationale of the procedures and the analysis. All authors participated in the preparation of the manuscript.
